# Curcumin inhibited the growth and invasion of human monocytic leukaemia SHI-1 cells *in vivo* by altering MAPK and MMP signalling

**DOI:** 10.1080/13880209.2019.1701042

**Published:** 2019-12-19

**Authors:** Guohua Zhu, Qun Shen, Hong Jiang, Ou Ji, Lingling Zhu, Linyang Zhang

**Affiliations:** a First Clinical College, Nanjing University of Chinese Medicine, Nanjing, China; b Department of Hematology, First Affiliated Hospital of Nanjing University of Chinese Medicine, Nanjing, China

**Keywords:** Acute myeloid leukaemia, metastasis, matrix metalloproteinase, mitogen-activated protein kinase

## Abstract

**Context:**

Curcumin, a polyphenolic compound extracted from the rhizome of the tropical plant *Curcuma longa* L. (Zingiberaceae), has been considered as a cancer chemopreventive drug by American National Cancer Institute.

**Objective:**

To examine the effect of curcumin on acute monocytic leukaemia SHI-1 cells *in vivo*.

**Materials and methods:**

The SHI-1 cells (1 × 10^6^ cells in 0.1 mL PBS) were injected subcutaneously into the right flanks of the female SCID mice. Curcumin dissolved in olive oil (15 and 30 mg/kg) was administered (i.p.) to mice once a day for 15 days while the control group received olive oil injection. Tumour proliferation and apoptosis were examined by PCNA, TUNEL and cleaved caspase-3 staining. The expression of MAPK, NF-κB, MMP9, MMP2 and vimentin were confirmed by RT-PCR, immunohistochemistry or western blotting.

**Results:**

Administration of curcumin significantly inhibited tumour growth, as the tumour weight decreased from 0.67 g (control) to 0.47 g (15 mg/kg) and 0.35 g (30 mg/kg). Curcumin inhibited the expression of PCNA and increased the degree of TUNEL and cleaved caspase-3 staining in tumour tissue. The results of western blotting showed that curcumin treatment inhibited NF-κB and ERK signalling while activating p38 and JNK. Moreover, curcumin attenuated the mRNA transcription and protein expression of MMP2 and MMP9. Curcumin also suppressed the level of vimentin.

**Discussion and conclusions:**

Our study demonstrates that curcumin can inhibit the growth and invasion of human monocytic leukaemia *in vivo*, suggesting the possible use of curcumin for anti-metastasis in leukaemia and the value of determining its unique target.

## Introduction

Acute myeloid leukaemia (AML) is a heterogeneous haematologic malignancy characterized by the clonal expansion of immature myeloid cells and bone marrow failure (Saultz and Garzon 2016). The complete remission rates of acute monocytic leukaemia are relatively low in the clinic, and a considerable number of patients die due to chemotherapy drug resistance as well as intramedullary and extramedullary relapse. AML is the most common form of acute leukaemia among adults, and it accounts for the largest number of annual deaths from leukaemia in the United States. An estimated 21,450 people are expected to be diagnosed with AML, and 10,920 patients are expected to die of AML in 2019 (Siegel et al. 2019).

In recent years, researchers have made great progress in understanding the pathogenesis and treatment of AML (Khan et al. 2017). To date, therapies used to treat AML have included cytotoxic agents as well as differentiation therapy such as all-*trans*-retinoic acid (ATRA). These therapeutic approaches have limited efficacy and may induce significant toxicity (Ryan 2018). Therefore, novel therapeutic agents with fewer toxic effects are urgently needed for AML therapy (Abedin and Altman 2016; Tamamyan et al. 2017).

Curcumin is a polyphenolic compound extracted from the dietary spice turmeric, which is now used as a spice, in food colouring, and as a traditional herbal medicine (Tsuda 2018). In addition to its antioxidant and anti-inflammatory action, curcumin has been reported to have good efficacy in cancer treatment, with various mechanisms and capabilities for targeting multiple cancer cell lines (Tomeh et al. 2019). For example, an anticancer effect of curcumin was reported to downregulate the expression of phosphatase of regenerating liver-3 (PRL-3) in highly metastatic melanoma cells (Wang et al. 2009). Curcumin also induced apoptosis associated with stress conditions in the endoplasmic reticulum in human papillary thyroid carcinoma as well as human liposarcoma (Wang et al. 2011; Zhang et al. 2018). Curcumin caused cytotoxicity that was dependent on the accumulation of reactive oxygen species in non-small-cell lung cancer cells (Wang et al. 2019). Apoptosis of mouse myeloma cells was induced by curcumin via the Notch3-p53 signalling axis (Zhang et al. 2019). Moreover, curcumin inhibits liver metastasis of gastric cancer by reducing circulating tumour cells (Gu et al. 2019). Curcumin inhibited SMADs signalling in TGF-β-dependent breast cancer cells metastasized to bone (Kunihiro et al. 2019). Curcumin attenuated hyperglycemia-driven EGF-induced invasive and migratory pancreatic cancer cells via suppression of the ERK and AKT pathways (Li et al. 2019). Angiogenesis in colorectal cancer was suppressed by curcumin via induction of oxidative stress (Moradi-Marjaneh et al. 2019). Curcumin suppressed hepatic stellate cell-induced hepatocarcinoma angiogenesis and invasion by downregulating connective tissue growth factor (Shao et al. 2019). Clinical trials indicate that curcumin is quite safe and exhibits therapeutic efficacy in patients with progressive, advanced cancers (James et al. 2015; Mahammedi et al. 2016; Pastorelli et al. 2018).

SHI-1 is an acute monocytic leukaemia cell line that was originally derived from the mononuclear cells of the bone marrow of a 37-year-old male with M5b in relapse and has been maintained as a stable cell line *in vitro*; it has an abnormal t (6; 11) (q27; q23) translocation and expresses the pathogenic fusion product of MLL/AF6 (Chen et al. 2005; Chen et al. 2007). Previously, we showed that curcumin could inhibit the proliferation and induce the apoptosis of SHI-1 cells *in vitro* (Zhu et al. 2016). In the present study, we confirmed that curcumin significantly induced apoptosis and partially suppressed the invasion of SHI-1 cells *in vivo*. The results provide a theoretical basis for future basic research on and clinical treatment of AML.

## Materials and methods

### Cells and main reagents

The SHI-1 cell line was a kind gift from the Jiangsu Institute of Haematology, and the use of this cell line was approved by the ethics committee of Nanjing University of Chinese Medicine. (The STR file of this line was provided in the Supplementary Material). The SHI-1 cells were incubated with IMDM medium (Gibco, Grand Island, NY) containing 15% foetal bovine serum (Gibco, Grand Island, NY) at 37 °C in a 5% CO_2_ incubator. Curcumin (S1848) was purchased from Selleck (Shanghai, China). TRIzol (RNAiso plus, 9109), the RNA reverse transcription kit (PrimeScript™ RT reagent Kit, RR037A) were purchased from TaKaRa Biotechnology (Dalian, China). The SYBR green fluorescent quantitative PCR mix (iQ™ SYBR1 Green Supermix) was bought from Bio-RAD (Bio-RAD, CA, USA). A GTVisin™ anti-mouse/anti-rabbit immunohistochemical analysis KIT was purchased from Gene Company (GK5005, GeneTech, Shanghai, China); TUNEL assay kits were purchased from Vazyme Biotech Co., Ltd. (Nanjing, China); PCNA (sc-56, 1:200 for IHC), vimentin (sc-373717, 1:100 for IHC), MMP9 (sc-21733, 1:200 for IHC, 1:500 for WB), MMP2 (sc-53630, 1:200 for IHC, 1:500 for WB), and β-actin (sc-47778, 1:2000 for WB) were purchased from Santa Cruz Biotechnology (Santa Cruz, CA, USA); ERK (#4695, 1:1000 for WB), JNK (#9252, 1:500 for WB), p38 (#8690, 1:2000 for WB), p65(#8242, 1:1000 for WB), p-ERK (#4370, 1:1000 for WB), p-JNK(#9255, 1:500 for WB), p-p38 (#4511, 1:1000 for WB), p-p65(#3033, 1:500 for WB) and cleaved caspase-3(#9664, 1:1000 for WB), were purchased from Cell Signalling Technology (Beverly, MA, USA). All other chemicals were obtained from Sigma-Aldrich (St. Louis, MO, USA).

### Animals

Female SCID mice on BALB/c background (BALB/cJGpt-*Prkdc^em1Cd561^*/Gp, 6–8 weeks old) were purchased from the Model Animal Research Centre of Nanjing University (Nanjing, China). The mice were housed in plastic cages at 21 ± 2 °C on a 12 h light-dark cycle with free access to food pellets and water. Animal welfare was ensured and all experiments were conformed to National Institutes of Health Guide for the Care and Use of Laboratory Animals. Experimental procedures were adhered to the Animal Care and Use Committee of Nanjing University of Chinese Medicine. The animal experiments were approved by the animal care and use committee of Nanjing University of Chinese Medicine. All efforts were made to minimize animal suffering and to reduce the number of animals used.

### Tumour growth assay *in vivo*

**Figure 1.**
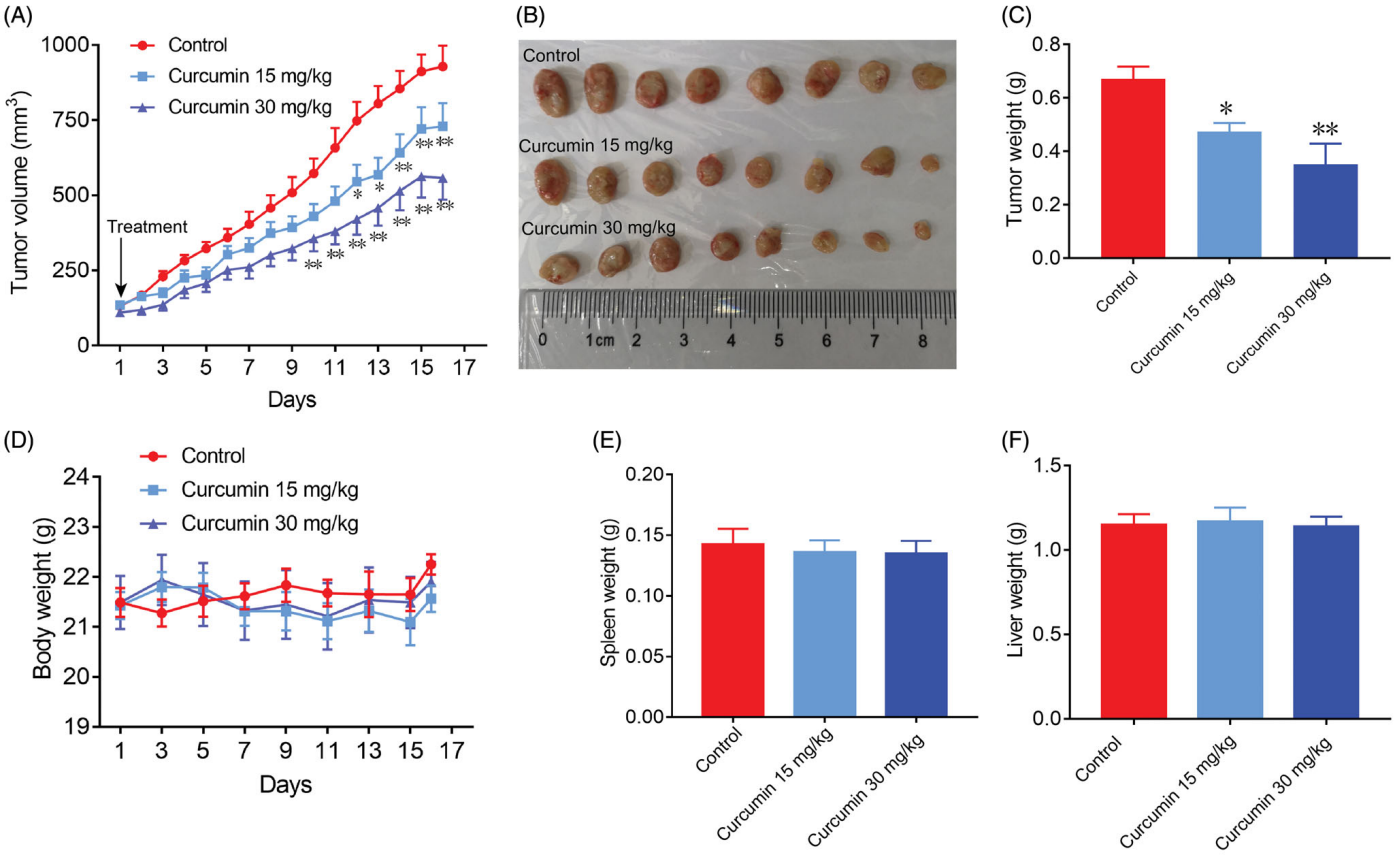
Curcumin reduced tumour growth *in vivo*. SHI-1 cells (1 × 10^6^) were transplanted subcutaneously into the right flank of SCID mice. Three days after transplantation, the mice were allocated to either the control or treatment groups, with 8 mice per group and this day was set as day one. Drugs were intraperitoneally injected started from day one as indicated in (A) (see Materials and methods section). (A) Tumour volumes were measured every day. After 16 days of treatment, the mice were sacrificed, and the solid tumours were separated, photographed (B) and weighed (C). (D) The body weight of the mice was measured every two days. The weights of the spleens and livers were recorded (E, F). Data are shown as the means ± SEM. *n* = 8. *p < 0.05, **p < 0.01 vs. the control.

The SHI-1 cells were cultured, collected by centrifugation (1000 rpm for 5 min) and washed twice with ice-cold PBS. They were then diluted to 1 × 10^7^ cells/mL with PBS, and 0.1 mL was injected subcutaneously into the right flanks of the female SCID mice. All mice formed tumours three days after injection. The mice were subsequently distributed into three groups randomly with eight mice per group and this day was set as day one. Curcumin was dissolved in olive oil (15 and 30 mg/kg) and administered (i.p., 100 μL) to each mouse every day from day 1 until day 15 as indicated in Figure 1(A). Tumour length and width were measured with a Vernieper every day, and tumour volume was calculated using the following equation: volume = a×b^2^/2, where a is the maximal width, and b is the maximal orthogonal length. On the 16th day, the mice were weighed and euthanized (cervical dislocation), and the tumours were removed and weighed.

### Real-time quantitative PCR

RNA was extracted from frozen tumour tissue using TRIzol reagent. All RNA samples were reverse transcribed individually using a reverse transcription kit according to the manufacturer’s instructions (PrimeScript™ RT reagent Kit, RR037A). The reaction volume was 20 μL containing: 1 μg RNA, 0.5 μL Oligo dT, 0.5 μL PrimeScript RT Enzyme Mix I, 0.5 μL dNTP, 2 μL 5 × PrimeScript buffer and RNase Free dH_2_O. The cDNA were subjected to quantitative PCR, which was performed with the BioRad CFX96 Touch™ real-time PCR detection system (BioRad, USA) using iQ™ SYBR1 Green Supermix, and threshold cycle numbers were obtained using BioRad CFX manager software. The reaction volume was 20 μL containing: 1 μL cDNA, 10 μL qPCR mix, 1 μL primers (forward and reverse), 8 μL RNase Free dH_2_O. The programme for amplification was 1 cycle of 95 °C for 2 min followed by 40 cycles of 95 °C for 10 s, 60 °C for 30 s, and 95 °C for 10 s. The primers used in this study were synthesized by GenScript Biotech Corporation (Nanjing, China) according to the sequences as follows:

GAPDH forward 5′-AGGGCTGCTTTTAACTCTGGT-3′
GAPDH reverse 5′-CCCCACTTGATTTTGGAGGGA-3′
MMP9 forward 5′-TTGACAGCGACAAGAAGTGG-3′
MMP9 reverse 5′-GCCATTCACGTCGTCCTTAT-3′
MMP2 forward 5′-TCTCCTGACATTGACCTTGGC-3′
MMP2 reverse 5′-CAAGGTGCTGGCTGAGTAGATC-3′

Reactions were run using GAPDH as the internal RNA control. The control samples were assigned as 1 and treated samples were described as percentage according to controls.

### Western blot analysis

Proteins from frozen tumour tissues were extracted in lysis buffer (30 mM Tris at pH 7.5, 150 mM sodium chloride, 1 mM phenylmethylsulfonyl fluoride, 1 mM sodium orthovanadate, 1% Nonidet P-40, 10% glycerol and phosphatase and protease inhibitors). Equal amounts of extracted proteins were denatured and subjected to SDS-PAGE. After electrophoresis and membrane transfer, the PVDF membranes (Millipore, Billerica, MA) containing the proteins were blocked with 5% BSA at room temperature (RT) for 2 h, incubated with the primary antibody at 4 °C overnight and then incubated with a horseradish peroxidase-coupled secondary antibody. Detection was performed using the LumiGLO chemiluminescent substrate system (KPL, Gaithersburg, MD, USA). The band intensity was measured by using Image J software, and the expression of proteins was normalized to the loading control (β-actin) was calculated.

### TUNEL assay

The TUNEL assay was performed according to the instructions provided by Vanzyme (A111, Nanjing, China). Briefly, the paraffin-embedded tumour sections were deparaffinized by xylene, rehydrated through an ethanol gradient (100, 90, 80 and 70%) and washed in PBS. After treatment with proteinase K (20 µg/mL) for 30 min, the sections were stained with TUNEL-FITC (1: 200) and then counterstained with DAPI for 10 min. Images were acquired by fluorescence microscopy (IX61, Olympus, Tokyo, Japan).

### Histopathological examination and immunohistochemistry

Removed tumour tissues were immersed in 4% paraformaldehyde for 48 h, embedded in paraffin. Histopathological study was performed using haematoxylin and eosin (H&E) stain. Paraffin-embedded tumour sections were heat-fixed, deparaffinized, rehydrated, antigen retrieval, blocked with 3% goat serum and incubated with primary antibodies overnight at 4 °C. Then the slides were detected using Real Envision Detection kit (GeneTech, Shanghai, China) according to the manufacturer’s instructions. The quantification of immunohistochemistry was performed using Plugins called IHC Profiler (https://sourceforge.net/projects/ihcprofiler). The score was divided into four levels (4: High Positive, 3: Positive, 2: Low Positive, 1: Negative). Acquired data were expressed as a histogram of mean ± SEM of three samples (five fields for each sample) in every group.

### Statistical analysis

Results were expressed as mean ± SEM of three independent experiments. Statistical analysis was performed with GraphPad Prism 7.0 software (San Diego, CA, USA). One-way ANOVA analysis followed by Dunnett’s *post hoc* test was used to evaluate the differences between various experimental and control groups when there were more than two groups. Student’s *t*-test was used to determine the significance of difference between two groups. *p* < 0.05 were considered as statistically significant.

## Results

### Curcumin inhibited SHI-1 cell proliferation *in vivo*

To confirm whether curcumin can inhibit the growth of SHI-1 cell proliferation *in vivo*, the effects of curcumin on the tumour growth of SHI-1 *in vivo* were tested with a subcutaneous xenograft model. Curcumin was administered once a day via i.p. injection to generate a dose-dependent inhibition of tumour growth in the tumour model examined (Figure 1(A–C)). Statistically significant (Student’s *t*-test) growth inhibition was obtained with both 15 and 30 mg/kg/day curcumin in the tumour xenografts tested. The tumour weight decreased from 0.67 g (control group) to 0.47 g (15 mg/kg group, *p* = 0.030) and to 0.35 g (30 mg/kg group, *p* = 0.003). Specifically, there was a 29.9% and 47.8% reduction in tumour weight observed in the 15 and 30 mg/kg/day curcumin-treated groups, respectively. Moreover, curcumin did not cause weight loss in the animals or decrease the spleen (*p* = 0.700 for 15 mg/kg group, *p* = 0.653 for 30 mg/kg group) and liver weights (*p* = 0.612 for 15 mg/kg group, *p* = 0.740 for 30 mg/kg group) (Figure 1(D–F)), indicating no significant adverse effects.

**Figure 2.**
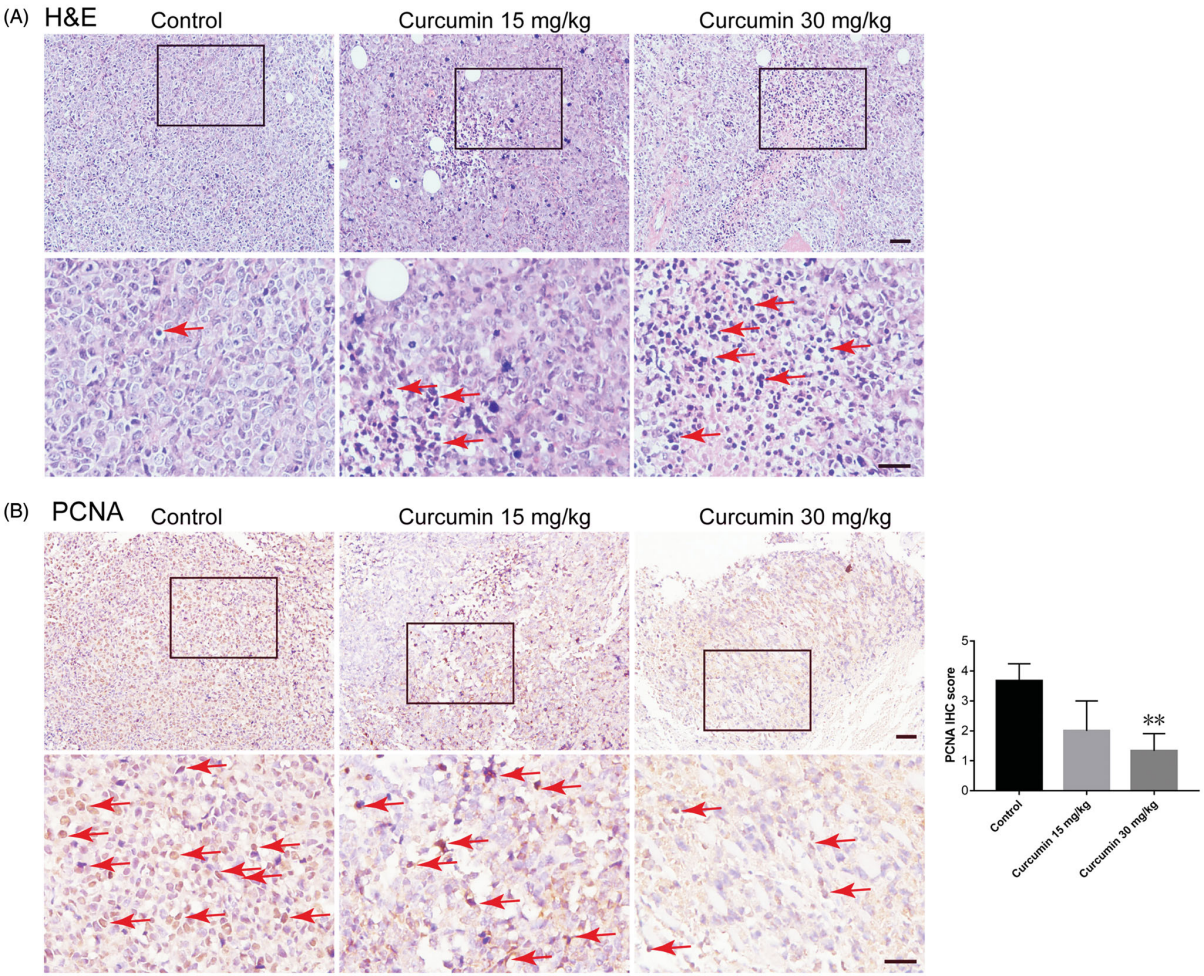
Curcumin inhibited tumour proliferation *in vivo*. (A) Paraffin sections of tumour tissues from mice were analysed by H&E staining. (B) The expression of PCNA in tumour tissue sections was assessed by immunohistochemistry staining. Scale bar: 50 μm. The images are representative of three samples per group, and quantified data are the average of three samples (five fields for each sample). Data are shown as the means ± SEM. *n* = 3. *p < 0.05, **p < 0.01 vs. the control.

Furthermore, the antitumor efficacy of curcumin was confirmed by H&E staining (Figure 2(A)). The results showed a different cellular architecture and typical pathological characteristics of malignancy compared to those observed in resected specimens from control mice with SHI-1. The curcumin-treated group showed massive cancer cell damage, such as condensation of the cytoplasm and pyknosis of nuclei (indicated by red arrows). Immunohistochemical staining of PCNA showed that curcumin treatment significantly inhibited SHI-1 cell proliferation *in vivo* (indicated by red arrows. *p* = 0.0668 for 15 mg/kg group and *p* = 0.0078 for 30 mg/kg group) (Figure 2(B)). These data indicated that curcumin reduced the tumour volume and growth rate of SHI-1 *in vivo*.

### Curcumin induced SHI-1 cell apoptosis in vivo

**Figure 3.**
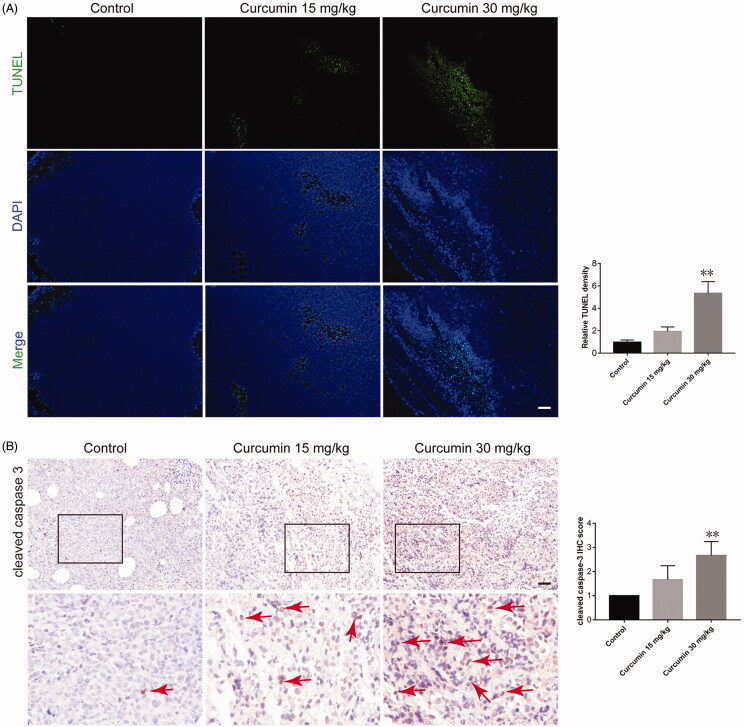
Curcumin enhanced tumour apoptosis. Paraffin sections of tumour tissues from mice were analysed by TUNEL staining (A) and cleaved caspase-3 staining (B). The images are representative of three samples per group, and quantified data are the average of three samples (five fields for each sample). Data are shown as the means ± SEM. *n* = 3. *p < 0.05, **p < 0.01 vs. Control.

TUNEL staining showed that green fluorescence was markedly elevated in tumour sections from the 30 mg/kg curcumin-treated group, implying that tumour cells underwent significant apoptosis after 30 mg/kg curcumin treatment (Figure 3(A)). Curcumin treatment also markedly increased the positive staining of cleaved caspase 3 in the tumour tissue (indicated by red arrows. *p* = 0.116 for 15 mg/kg group and *p* = 0.0075 for 30 mg/kg group) (Figure 3(B)).

### Curcumin induced SHI-1 cell apoptosis by altering MAPK and inhibiting NF-κB

**Figure 4.**
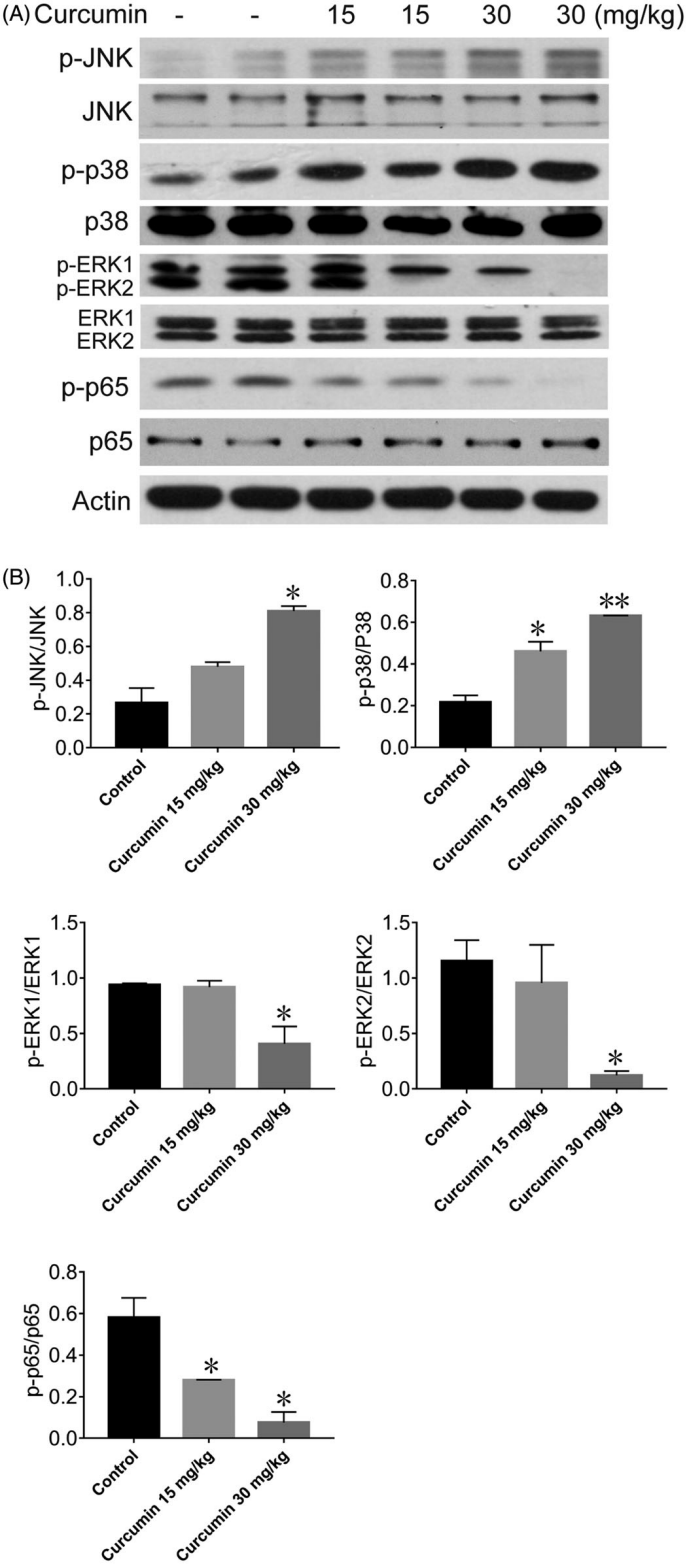
Effects of curcumin on p38, JNK, ERK and NF-κB expression in SHI-1 tumour tissue. (A) The protein from SHI-1 tumour tissue was extracted; the expression levels of p38, JNK, ERK and NF-κB were detected by western blotting; and the relative band intensity was calculated (B). Data are shown as the means ± SEM. *n* = 3. *p < 0.05, **p < 0.01 vs. the control.

As we previously reported, curcumin could induce SHI-1 cell apoptosis by affecting MAPK and inhibiting NF-κB signalling *in vitro* (Zhu et al. 2016). Thus, we next examined whether MAPK family members were involved in the curcumin-mediated apoptosis of SHI-1 cells *in vivo*. As shown in Figure 4, the phosphorylation levels of p38 (38 kDa) (*p* = 0.0439 for 15 mg/kg group and *p* = 0.0059 for 30 mg/kg group) and JNK (46 and 54 kDa) (*p* = 0.0823 for 15 mg/kg group and *p* = 0.0145 for 30 mg/kg group) were increased after curcumin treatment, while the phosphorylation of ERK1 (44 kDa) (*p* = 0.774 for 15 mg/kg group and *p* = 0.0475 for 30 mg/kg group) and ERK2 (42 kDa) (*p* = 0.662 for 15 mg/kg group and *p* = 0.0372 for 30 mg/kg group) were decreased.

As shown in Figure 4, the expression of the p-p65 (65 kDa) protein was reduced by curcumin treatment in a dose-dependent manner (*p* = 0.048 for 15 mg/kg group and *p* = 0.0427 for 30 mg/kg group). Therefore, curcumin-induced apoptosis of SHI-1 cells was likely also related to the inhibition of NF-κB activity.

### Curcumin decreased the expression levels of MMP2, MMP9 and vimentin

**Figure 5.**
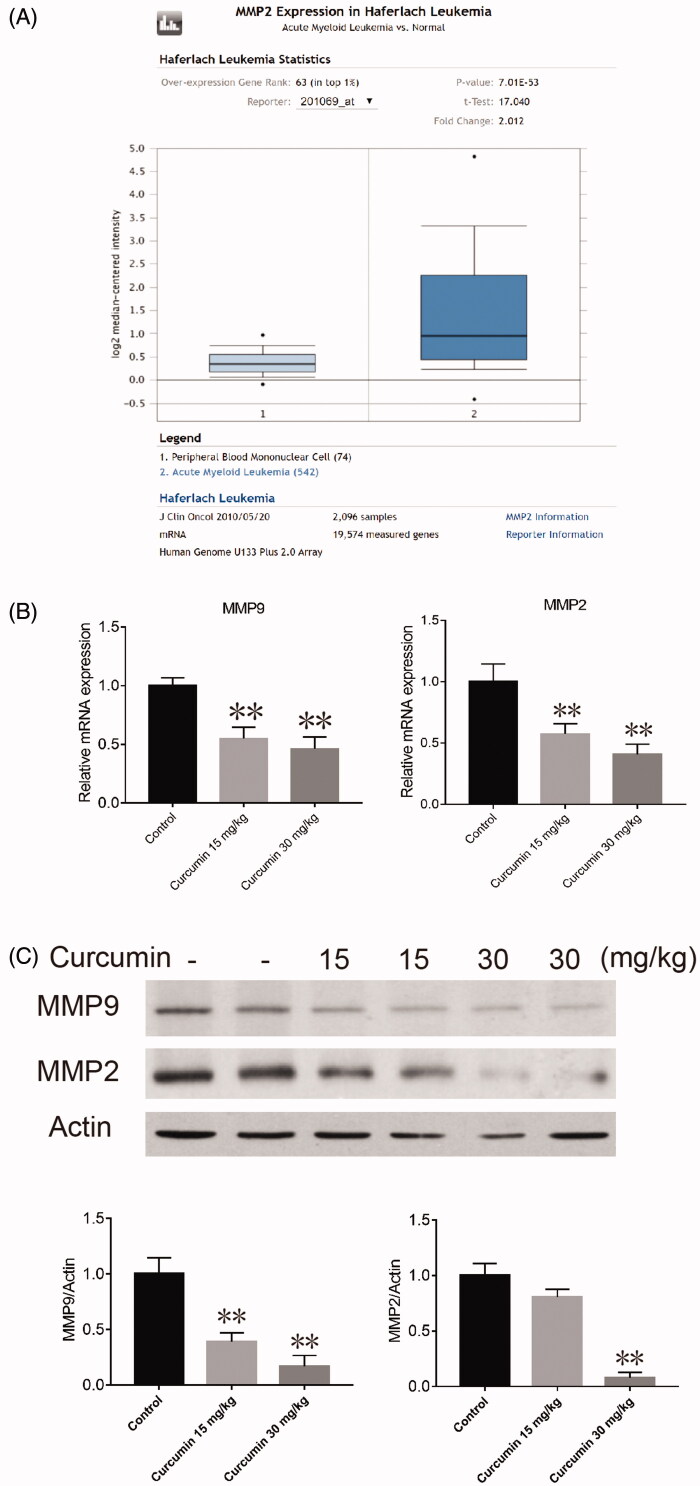
Effect of curcumin on MMP9 and MMP2 gelatinase levels in SHI-1 tumour tissue. (A) Differential analysis of MMP2 comparing AML and normal tissue with the Oncomine database. (B) The mRNA from SHI-1 tumour tissue was extracted, and the expression levels of MMP9 and MMP2 were detected by RT-PCR. (C) The protein from SHI-1 tumour tissue was extracted; the expression levels of MMP9 and MMP2 were detected by western blotting; and the relative band intensity was calculated. Data are shown as the means ± SEM. *n* = 3. *p < 0.05, **p < 0.01 vs. the control.

**Figure 6.**
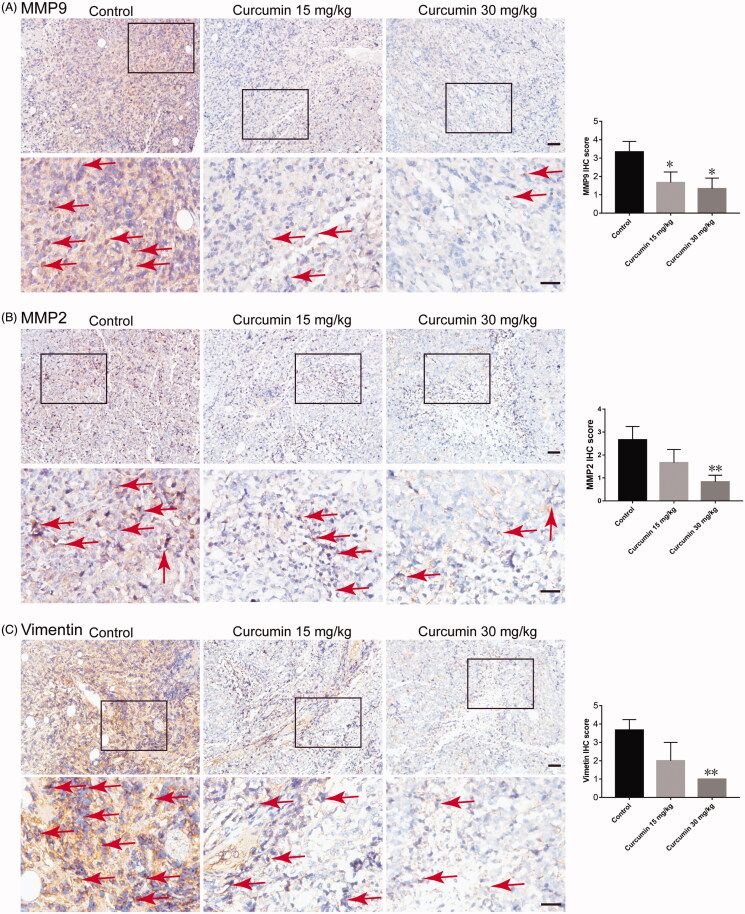
Curcumin reduced MMP9 and MMP2 and vimentin expression in tumour tissue. The expression of MMP9, MMP2 and vimentin in tumour tissue sections was assessed by analysis of the immunohistochemistry staining. The images are representative of three samples per group, and quantified data are the average of three samples (five fields for each sample). Data are shown as the means ± SEM. *n* = 3. *p < 0.05, **p < 0.01 vs. the control. Scale bar: 50 μm.

In addition to the anti-proliferative and apoptosis-inducing abilities of curcumin, we also wondered whether curcumin could affect the metastatic potency of SHI-1 cells *in vivo*. Through differential analysis of MMP2 in AML and normal tissue using the Oncomine database, we confirmed that the expression levels of MMP2 was higher in AML than in normal peripheral blood (Figure 5(A)). The results of real-time quantitative PCR and western blot analysis showed that the mRNA and protein expression of MMP9 (mRNA*: p* = 0.035 for 15 mg/kg group and *p* = 0.0215 for 30 mg/kg group. Protein (92 kDa): *p* = 0.035 for 15 mg/kg group and *p* = 0.0215 for 30 mg/kg group) and MMP2 (mRNA*: p* = 0.035 for 15 mg/kg group and *p* = 0.0215 for 30 mg/kg group. Protein (72 kDa): *p* = 0.168 for 15 mg/kg group and *p* = 0.0081 for 30 mg/kg group) in SHI-1 tumour tissue was decreased after curcumin treatment (Figure 5(B,C)). Further, immunohistochemistry staining also suggested a decrease in the level of MMP9 (indicated by red arrows. *p* = 0.021 for 15 mg/kg group and *p* = 0.0132 for 30 mg/kg group) and MMP2 (indicated by red arrows*. p* = 0.101 for 15 mg/kg group and *p* = 0.0079 for 30 mg/kg group) (Figure 6(A)).

Vimentin is a type III intermediate filament that maintains cell integrity and is involved in cell migration, motility and adhesion. When overexpressed in solid cancers, vimentin drives EMT and, ultimately, metastasis. Our results showed that the expression of vimentin in SHI-1 tumour tissue was significantly inhibited after curcumin treatment (indicated by red arrows. *p* = 0.067 for 15 mg/kg group and *p* = 0.0013 for 30 mg/kg group) (Figure 6(B)), indicating reduced metastatic potency of SHI-1.

## Discussion

Acute myeloid leukaemia (AML) is a complex hematological disease characterized by genetic and clinical heterogeneity. Recent advances in the understanding of AML pathogenesis have made possible the development of new agents that target specific molecules or mechanisms and contribute to efforts for moving beyond the current standard of care. In particular, new therapeutic options such as targeted therapies (midostaurin and enasidenib), monoclonal antibodies (gemtuzumab ozogamicin), and a novel liposomal formulation of cytarabine and daunorubicin (CPX-351) have been recently approved (Luppi et al. 2018). Currently, chemotherapy is still the main strategy used for AML treatment. Despite high response rates after initial chemotherapy in patients with AML, relapses occur frequently, resulting in a five-year survival rate of <30% of patients (Siegel et al. 2019). Several disease-specific and patient-specific factors, such as cytogenetic/molecular features, age, performance status, and comorbidities, often limit the use of high-dose cytotoxic chemotherapy (Ferrara and Schiffer 2013; Dohner et al. 2017). Thus, increasing attention has been paid to finding new strategies with high efficacy and low toxicity for AML therapy.

Thus far, combination therapy based on traditional cytotoxins plus personalized treatments seems to offer the best anti-leukemic effects and higher response rates with manageable toxic effects. An increasing number of patients select to use naturopathic therapy with traditional Chinese medicine (TCM) for adjuvant treatment (Nie et al. 2016). TCM treatments, including herbal compounds and herbal monomers, are proposed to work on multiple targets in whole-body systems to fight against the disease (Bailly 2009; Pan et al. 2011). For AML, studies have provided real-world data suggesting that all AML patients, regardless of age or other prognostic factors, may achieve longer survival by receiving TCM treatments in addition to standard therapy (Wang et al. 2016; Fleischer et al. 2017). In the present study, many methods have been employed to confirm the growth inhibition potency of curcumin on SHI-1 *in vivo*. A terminal deoxynucleotidyl transferase (TdT) dUTP nick-end labelling (TUNEL) assay was designed to detect cells that underwent extensive DNA degradation during the late stages of apoptosis. The method is based on the ability of TdT to label the blunt ends of double-stranded DNA breaks independent of a template (Kyrylkova et al. 2012; Crowley et al. 2016). As the primary executioner of programmed cell death, caspase 3 is directly or indirectly responsible for the cleavage of many proteins and caspases involved in apoptosis (Elmore 2007). All the results demonstrated that apoptosis induction by curcumin contributed to the growth inhibition of SHI-1 *in vivo*.

Several studies have provided evidence for the effect of curcumin on AML. For example, curcumin could enhance the cytogenotoxic effect of etoposide in HL-60 cells through induction of reactive oxygen species (Papież et al. 2016). Curcumin sensitizes acute promyelocytic leukaemia cells to apoptosis induced by cellular responses to unfolded protein by suppressing the loss of misfolded N-CoR protein (Wang et al. 2011). Yu et al. (2013) reported that curcumin downregulates DNA methyltransferase 1 and plays an anti-leukemic role in acute myeloid leukaemia both *in vitro* and *in vivo*. Curcumin also blocks Kv11.1 (erg) potassium current and slows proliferation in the infant an acute monocytic leukaemia cell line THP-1 (Banderali et al. 2011). The expression of the Wilms’ tumour 1 gene was decreased by curcumin treatment, which upregulated miR-15a and miR-16-1 in leukemic cells, including HL-60 and K562 (Gao et al. 2012). SHI-1 is a new monocytic leukaemia cell line with t (6;11) translocation, p53 gene alterations, and high tumorigenicity in nude mice. SHI-1 could be a valuable tool in the study of leukemogenesis (Chen et al. 2005; Chen et al. 2007). Together with our previously reported results *in vitro*, these findings show that curcumin has very good capacity for inhibiting SHI-1 tumour both *in vivo* and *in vitro*, and it was well tolerated in the mice, suggesting the possible use of curcumin against AML in the clinic.

In addition to its malignant proliferation property, according to scientists, leukaemia is endowed with inherent metastatic potential (Trendowski 2015). The metastatic process comprises a complex series of steps in which cancer cells leave the original site and migrate to other parts of the body. In the case of leukaemia, cells acquire the ability to penetrate the walls of blood vessels of other tissues in the body and engaging cells at this new site, where it eventually forms another clinically detectable tumour known as a metastatic or secondary tumour implant (Massague and Obenauf 2016; Lambert et al. 2017). Although rare, the metastasis of blood and lymphatic system cancers to the lung, heart, central nervous system, and other tissues has been reported (Facenda-Lorenzo et al. 2016; Meena et al. 2018). As reported, epithelial-mesenchymal transition (EMT) transcription factors are considered potentially important prognostic or predictive factors of leukaemia and targets for leukaemia treatments (Chen et al. 2018). Moreover, the overexpression of the EMT marker vimentin is associated with poor clinical outcome in patients with AML (Wu et al. 2018). Over the past decade, it has become evident that, to colonize new tissue, leukaemia cells need to develop a premetastatic niche via extracellular matrix remodelling, which they achieve by releasing MMPs (Shay et al. 2015). Studies have confirmed that metalloproteinases, including MMP2 and MMP9, play an important role in the invasion process of various solid tumours by digesting the extracellular matrix barrier (Deryugina and Quigley 2006; Chaudhary et al. 2016). Thus in order to minimize treatment-associated mortality, suppression of EMT as well as expression of metalloproteinases may represent a rational alternative strategy for reducing metastatic potential of vimentin. As our results showed, SHI-1 cells expressed high levels of MMP9, MMP2 and vimentin, while curcumin treatment reduced the levels of both of them, indicating that curcumin may contribute to the prevention of AML metastasis.

Collectively, our study provided evidence *in vivo* to confirm the effect of curcumin on the apoptosis and invasion of human acute leukaemia SHI-1 for the first time. Curcumin activated JNK and p38 but inhibited ERK and NF-κB signals, which resulted in SHI-1 tumours of reduced volume. Furthermore, curcumin may also downregulate the expression of MMP9 and MMP2 as well as vimentin, leading to suppression of the metastatic potency of SHI-1. Our data suggest that curcumin may be a promising candidate for AML therapy. Furthermore, several issues need to be clarified in our next study, including the detailed target for curcumin on SHI-1 as well as its possible effect on the differentiation of AML.
